# Effect of Extrusion Screw Speed and Plasticizer Proportions on the Rheological, Thermal, Mechanical, Morphological and Superficial Properties of PLA

**DOI:** 10.3390/polym12092111

**Published:** 2020-09-16

**Authors:** Jaime Gálvez, Juan P. Correa Aguirre, Miguel A. Hidalgo Salazar, Bairo Vera Mondragón, Elizabeth Wagner, Carolina Caicedo

**Affiliations:** 1 Grupo de Investigación en Desarrollo de Materiales y Productos—GIDEMP, Centro Nacional de Asistencia Técnica a la Industria—ASTIN, SENA, Calle 52 No 2bis 15, Cali 760035, Colombia; jagalvez39@misena.edu.co (J.G.); bvera@sena.edu.co (B.V.M.); ewagner@misena.edu.co (E.W.); 2 Research Group for Manufacturing Technologies (GITEM), Universidad Autónoma de Occidente, Cali 760035, Colombia; jpcorrea@uao.edu.co (J.P.C.A.); mahidalgo@uao.edu.co (M.A.H.S.); 3 Grupo de Investigación en Química y Biotecnología (QUIBIO), Facultad de Ciencias Básicas, Universidad Santiago de Cali, Calle 5 No. 62-00, Cali 760035, Colombia

**Keywords:** biopolymers, acetyl tributyl citrate, poly(lactic acid), dynamic mechanical analysis, contact angle

## Abstract

One of the critical processing parameters—the speed of the extrusion process for plasticized poly (lactic acid) (PLA)—was investigated in the presence of acetyl tributyl citrate (ATBC) as plasticizer. The mixtures were obtained by varying the content of plasticizer (ATBC, 10–30% by weight), using a twin screw extruder as a processing medium for which a temperature profile with peak was established that ended at 160 °C, two mixing zones and different screw rotation speeds (60 and 150 rpm). To evaluate the thermo-mechanical properties of the blend and hydrophilicity, the miscibility of the plasticizing and PLA matrix, Fourier transform infrared spectroscopy (FT-IR), thermogravimetric analysis (TGA) and differential scanning calorimetry (DSC), oscillatory rheological analysis, Dynamic Mechanical Analysis (DMA), mechanical analysis, as well as the contact angle were tested. The results derived from the oscillatory rheological analysis had a viscous behavior in the PLA samples with the presence of ATBC; the lower process speed promotes the transitions from viscous to elastic as well as higher values of loss modulus, storage modulus and complex viscosity, which means less loss of molecular weight and lower residual energy in the transition from the viscous state to the elastic state. The mechanical and thermal performance was optimized considering a greater capacity in the energy absorption and integration of the components.

## 1. Introduction

Nowadays, there is a great need to resolve problems associated with the high consumption of non-renewable resources, to produce non-degradable polymers, which is generating a high amount of plastic waste [1]; biodegradable aliphatic polymers, such as poly-hydroxyalkanoates (PHAs), poly-butylene succinate (PBS), poly-butylene succinate-co-adipate (PBSA), poly(butylene adipate-co-terephthalate) (PBAT) or poly-lactic acid (PLA), are an important alternative [2,3,4]. Among these biopolymers, PLA is the key and promising biopolymer to develop new materials both in industry and in academy [5], commercially is the most traded and it is obtained from the fermentation of renewable sources such as whey, corn, potato, molasses, or sugar feed stocks to produce the monomer lactic acid [6,7], which is subsequently polymerized to a linear aliphatic thermoplastic polyester by a ring-opening synthesis procedure [8].

PLA possesses interesting features, such as sustainability, relatively easy processing, the capacity for heat sealing, high transparency, gloss, printing ability [2]; moreover, it has excellent optical properties and high tensile strength but, unfortunately, it is rigid and brittle [9]. Therefore, the PLA presents low flexibility, low impact resistance, poor thermal properties during processing and low crystallization rates [10,11,12], which hinder their uses, such as packaging or textile fibers; thus, it is necessary to use a plasticizer to improve its processability, flexibility, break elongation, and impact properties. However, not all the plasticizers are suitable because for food packaging, there are some requirements related with the contact area and migration [13]. The plasticization process should, at the optimal percentage of the biocompatible plasticizer, be compatible with the PLA, stable at the processing temperatures, reduce the glass transition of the polymeric amorphous domain, reducing the melting point of the crystalline phase, all this to enhance the molecular mobility of the PLA chains [9,14,15,16,17].

Several plasticizers have been used to improve the undesirable processing properties of the PLA; they include low and high molecular weight plasticizers depending of the required mobility within the PLA matrix [14,18,19]. Some of the most high molecular weight plasticizers reported are polyethylene glycol (PEG), acrylated polyethyleneglycol (PEGA), lactic acid oligomer (LAO), citrate oligoesteres, and malonate oligomers. With PEG, the plasticizing efficiency increases with decreasing molecular weight, which leads to a decrease in PLA molecular weight, resulting in a decrease in the PLA viscosity [16,20]; the PEGA increases the crystallinity compared with neat PLA [8]; in the case of the OLA, a drastic phase separation and degradation of the mechanical properties was observed [21]. Because of this, the OLA has been used with PLA-based blends to reduce migration rate and detrimental changes in the material properties [22], showing migration levels below admitted levels, and no apparent phase separation was found due to a good compatibility between the polymeric matrix and the OLA; when citrate oligoesteres and malonate oligomers were blended with PLA, they increased the mobility, but the phase separation and migration phenomenon occurred. In addition, oligomer obtainment involves a synthesis step [15,23]. 

On the other hand, regarding the low molecular weight plasticizers, the cardanol (CD) is a liquid extracted from the cashew nut (30% content) and has been studied as plasticizer finding good plasticizing efficiency decreasing the glass transition temperature and enhanced the crystallization ability of PLA [24]; while citrate esters as triethyl citrate (TEC) [25,26], tributyl citrate (TBC) [14] and acetyl tributyl citrate (ATBC) [6,13,16,25,27] are plasticizers widely studied, some studies have focused their analysis on the process conditions for film extrusion and welding of biodegradable polymer films, finding that their addition significantly reduced the T_g_ of PLA, while the storage of the laminated films resulted in increased crystallinity because glass transition temperatures, around room temperature, allowed the reorganization of the polymer chains and lead to migration of plasticizers within the PLA matrix due to their high mobility [28]. This citrate plasticizers migrated to the film surface after storage, and appeared to cause an increase in surface crystallinity and hydrophilicity [29]. Alternatives were reported to minimize migration, and phase separation was through functional group grafts that allowed esterification or increased interaction through hydrogen bridges; however, mechanical strength decreased, while T_g_ increased significantly [14]. Recently, it was found that ATBC exhibits a great plasticizing effect to the neat PLA, and the migration from the blend was noted at high temperatures and relatively long times during the migration test [25]; these results have been discussed from the molecular point of view based on factors such as miscibility and molecular weight, but a study focused on the shear conditions of the blend has not been found.

Concerning the influence of processing parameters on the properties of plasticized PLA, different blending conditions and systems have been used. The melt process is carried out by heating the material above its melting temperature to get the desired product [30] and also is necessary take into account during the blending process, the shearing conditions [7]. At lower processing speeds between 20 and 80 rpm, various blending systems were obtained from mixers or torque rheometers [8,9,13,24,25,26,31], or from twin screw extruders [14,16,20], using plasticizers such as ATBC, PEG or TBC, and PLA or a PLA-blend, varying only the plasticizer content in the final PLA-based blend; generally, for processing speeds above 100 rpm, twin screw extruders [1,2,6,7,10,15,22,23] are used to obtain the plasticized PLA-based blend material from ATBC, OLA, oligoesteres or malonate oligomers to plasticize the PLA or a PLA-blend. In the most of cases, especially with ATBC, the plasticizer decreases the glass transition temperatures and improve the ductile properties but there is always a migration of the plasticizer from the PLA matrix. So far, studies that allow us to know the effect of the extrusion speed in the plasticization process have not been found; nonetheless, it is important to know the effect that speed has on the compatibility of the plasticizer in the PLA matrix, the morphological stability of the plasticized material and the prevention of the migration of the plasticizer from the bulk material.

In this study, PLA was plasticized with ATBC by continuous mixing using a screw configuration with two effective mixing zones [32]. Our research studies the behavior of various materials at different processing speeds, analyzing the effects of molecular interactions according to thermal properties, as well as the rheological characteristics and physical parameters of the material during the processing.

## 2. Materials and Methods 

### 2.1. Materials

Ingeo 2003-D polylactic acid (PLA) from Nature Works Company (Lancaster, PA, USA) with a density of 1.24 g/cm³ and MFR (210 °C/2.16 kg) 6.0 g/10 min. Commercial grade acetyltributyl citrate (ATBC) with a density of 1.05 g/cm³ and purity < 99% was purchased from SUCROAL S.A (Cali, Colombia).

### 2.2. Preparation of Blends PLA/ATBC

The PLA was placed in an oven at 100 °C for 3 h; subsequently, 6 mixtures of plasticized PLA in a twin-screw extruder at different speeds (60 rpm and 150 rpm) were obtained from PLA and acetyl tributyl citrate (ATBC) as a plasticizer. Three different ATBC concentrations were selected 10, 20, and 30 wt%. For the experimental design, the samples were abbreviated as PLA*X*-*Y* with *X* = extrusion screw speed and *Y* = ATBC proportion is enough, for example, PLA60-20 is a PLA obtained at 60 rpm and containing 20% ATBC; PLA150-30 is a PLA obtained at 150 rpm and containing 30% ATBC, and finally PLA60-0 or PLA60 is a PLA obtained at 60 rpm, without the addition of plasticizer used as a target. 

**Figure 1:**
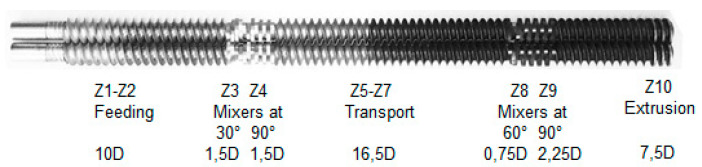
Screw configuration used in the study.

The mixtures of PLA with ATBC were made in a Thermo Scientific-Haake Rheomex OS PTW16 double screw spindle extruder in a co-rotating parallel configuration, with 16 mm diameter and 40D total length (see Figure 1, with zones distributed of transport (81.25%) and mixers (18.75%), located at 10D and 30D length, respectively) similar to that reported by Liao et al. [32]. Screw speeds of 60 and 150 rpm were used to optimize the material final properties, while the temperature profile was set up at 130, 140, 150, 160, 170, 180, 180, 190, 190, 180, and 160 in the ten different extrusion zones (Z*n*). The PLA is entered into the volumetric type feeding system (zone 1) and ATBC was added (zone 2) using a peristaltic pump.

From the extruded materials, sheets were made using compression molding. First, the materials were dried in a vacuum oven at 50 °C for at least 8 h. After this, the samples were pressure molded at between Teflon sheets at 30 bar and 180 °C for 5 min using a hot plate press with a forced water cooling system (LabPro 400, Fontijne Presses, Delft, Netherlands), which enables the controlled cooling of the sheets from the processing temperature to room temperature at a similar cooling rate. These similar cooling conditions for all materials enable comparisons of the different properties evaluated as shown in a previous research work reported earlier by our group [33,34]. The obtained sheets have a thickness of 2 mm and were used for tensile, Dynamic Mechanical Analysis (DMA) (company, city, country), Fourier transform infrared spectroscopy (FT-IR) (Agilent, SC, USA) and contact angle tests. These specimens were cut from the sheets using a water jet cutter (Protomax-Omax, Kent, USA ). As it is a fast and cold cutting procedure, it is the preferred method when the materials being cut (as PLA) are sensitive to the high temperatures generated by other methods.

### 2.3. Rheological Analysis

The viscosity was determined by a rotational rheometer (DHR-2, TA Instruments, USA) with controlled stress and the cone-plate configuration using the equilibrium flow test with a gap of 145 µm, a diameter of 25 mm and an angle of 5753°. The rheological measurements were performed at 160 °C and the shear rate was in the range of 0.001 to 300 s^−1^. The dynamo-mechanical test settings were: constant strain amplitude was maintained at 1% and the measurements were carried out in the linear viscoelastic regime with a frequency range of 0.05 to 627.99 Hz. Zero shear viscosity ($\eta_{0}$) was determined by the Carreau–Yasuda model fitted to the experimental data [35]. $\eta_{0}\;$ was possible due to the rheological measurements in oscillatory mode, and the calculations that were performed applying the Trios 5.0 software [7] describe pseudoplastic flow with asymptotic viscosities at zero and infinite shear rates, according to Equation (1) [35]:

(1) $$\eta \left(\gamma \right)=\eta_{0}{\left[1+{\left(\lambda \gamma \right)}^{a}\right]}^{\frac{n-1}{a}}$$

where *n* is a power-law coefficient, a is the adjustable exponent, γ is a shear rate, and *λ* is characteristic time. The theoretical value of the molecular weight (Mw) of the PLA plasticized at different speeds based on rheological data ($\eta_{0}$, Equation (1)) according Equation (2) was estimated:

(2) $$\log\eta_{0}\;=\;3.4\;\log\;\mathrm{Mw}\;-\;C(T)$$

where C(T) = 14.83, as revealed from literature data [36].

### 2.4. Fourier Transformed Infrared Spectroscopy (FTIR)

FTIR analysis of sheets was carried out in attenuated total reflection (ATR) mode with a Shimadzu IR Affinity-1. The spectra were collected from 16 scans at a resolution of 4 cm^−1^, the tests were performed at room temperature and a background spectrum was obtained before each test to compensate by spectra subtraction the humidity effect and the presence of carbon dioxide. The spectra were normalized using the C–H stretching peak at 2995 cm^−1^ as a reference.

### 2.5. Thermogravimetric Analysis (TGA) and Differential Scanning Calorimetry (DSC)

The TGA were performed on a TGA/DSC 2 STAR System thermogravimetric analyzer, Mettler Toledo, USA. The samples (10 ± 0.5 mg) were placed in alumina (Al_2_O_3_) crucibles at a heating rate of 20 °C min^−1^ using a nitrogen purge at a flow rate of 60 mL/min, and the analysis was done at a temperature range between 30 and 600 °C. DSC analyses were performed on a TA Instruments model Q-2000 machinewas done by sealing ~10 mg of the sample in aluminum pans and put through heating condition at a rate of 10 °C min^−1^. The samples were subjected to heating–cooling–heating cycles. They were firstly heated from −25 to 200 °C, then were cooled from 200 to 25 °C and were again heated from 25 to 200 °C. For the evaluation of thermal properties, the results of the second heating run were used because of the elimination of the thermal history of the test specimens. The calculations are based on ΔH_m_^0^ for 100% crystalline PLA being equal to 93 J/g [37]. The degree of crystallinity (X_c_) of the blends could be calculated from the melting enthalpy in the secondary heating curves (ΔH_m_) according to the following equation:

%X_c_ = [ΔH_m_ − ΔH_cc_ /ω_PLA_ · ΔH_m_^0^ ] ×100 (3)

where ω_PLA_ is the mass fraction of PLA in the blends and ΔH_cc_ is cold crystallization enthalpy. 

### 2.6. Tensile Properties

Tensile tests were performed according to the ASTM D 638-14 standard in a universal testing machine INSTRON, (Norwood, MA, USA) Model 3366 equipped with a 10 kN load cell. The tests were performed at 23 °C, using a constant rate of 10 mm/min and type V samples.

### 2.7. Dynamic Mechanical Analysis (DMA)

To study the viscoelastic behavior of the materials, the linear viscoelastic region (LVE) of the PLA were identified using a TA Instruments DMA RSA-G2 equipped with a three-point bending geometry (10 mm span) at a frequency of 1 Hz. For this study, strain sweeps from 0.001 to 0.1% were carried out at −50, 25 and 80 °C with a 1 Hz frequency. After this, temperature ramp tests were performed to observe the behavior of the blends at different temperatures. These tests were performed between −50 and 150 °C, at 1 Hz, 3 °C/min, and 0.01% of strain.

### 2.8. Scanning Electron Microscopy Analysis

A scanning electron microscope (SEM) JEOL, JCM 50000 (Tokyo, Japan) was used to scan the surface morphology of the tensile fracture surface samples. The samples were coated with a thin layer of gold before scanning observation in order to boost the sample conductivity. A voltage of 10 kV was applied and magnifications of 1700× and 500× were taken.

### 2.9. Contact Angle Analysis

To carry out this analysis, films of the PLA blends were obtained in a press at a temperature of 110 °C and a pressure of 2 tons. The contact angle was measured using a Ramé-Hart Model 250 goniometer with an optical system (Ramé-hart instrument co., Succasunna, NJ, USA) with which the interaction of water (2 μL) with the surface of the films of the samples was observed. The image was captured after 60 s and the analysis was carried out with Image J software. Three measurements were made for each sample to take an average of the measurements.

## 3. Results and Discussion

### 3.1. Rheological Analysis

**Figure 2:**
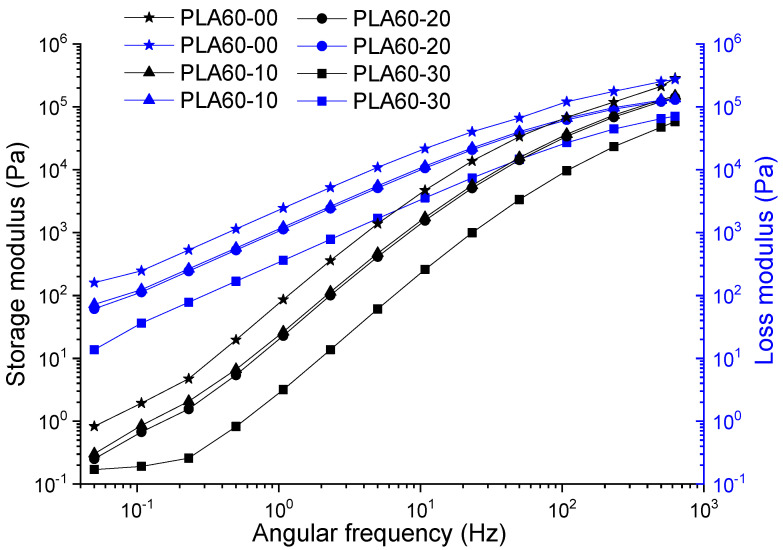
Storage and loss modulus of poly-lactic acid (PLA) and PLA with acetyl tributyl citrate (ATBC) at 10, 20 and 30% extruded to 60 rpm.

**Figure 3:**
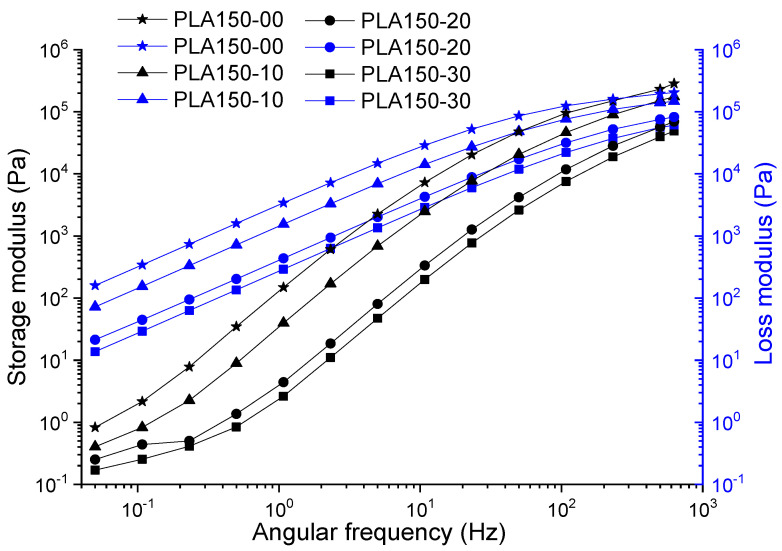
Storage and loss modulus of PLA and PLA with ATBC at 10, 20 and 30% extruded to 150 rpm.

The curves corresponding to the storage and loss modulus were analyzed according to the angular frequency for the target samples in this study (PLA, PLA: ATBC), which are presented in Figure 2 and Figure 3. In general, these exhibit an increase in the value of the modulus as the angular frequency increases, typical behavior of the polymers, as a consequence of the contribution of the energy necessary to produce an increase in the mobility of the polymer chains. PLA samples without plasticizer processed at 60 and 150 rpm (PLA60 and PLA150) showed viscous to elastic transitions at frequencies of 510 and 300 Hz, with moduli of 120 and 160 kPa, respectively. The increase in the proportion of plasticizer on the PLA at different extrusion speeds retards the transition (making liquid behavior more durable) and decreases the value of G′ from ~150 to ~14 Pa and G″ ~0.8 to ~0.1 Pa. The foregoing is consistent with the action presented by plasticizers as lubricating agents within the polymer, causing the polymer chains to slide smoothly. On the other hand, the slopes of the curves have similar behavior in the PLA at different extrusion speeds, the storage modulus presents an increase up to two orders of magnitude to intercept the loss modulus, which is evidenced in the range of studied frequency of the material between 0.1 and 630 Hz corresponding to the viscoelastic linear region where the deformation is reversible. In general, G″ and G′ decrease when the extrusion speed increases up to 20% of the plasticizer and 60 rpm. In this proportion, at higher speeds, the values of G′ with respect to G″ remain low; this due to the high molecular mobility and disentanglement of the polymer chains.

**Figure 4:**
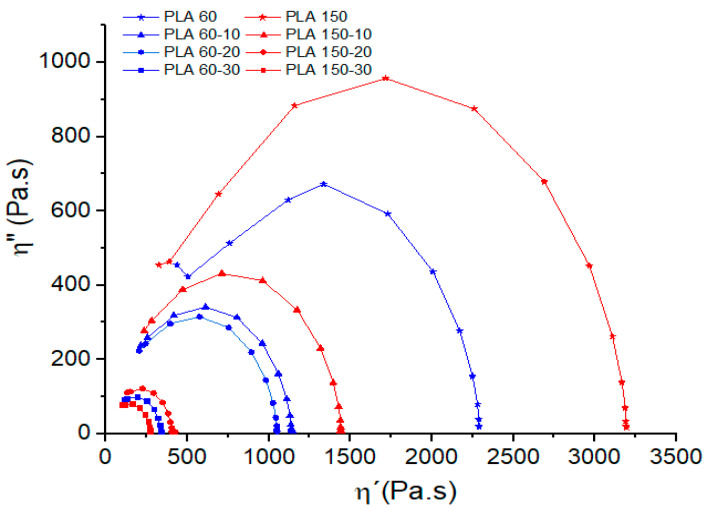
Cole–Cole diagrams of PLA and PLA with ATBC at 10, 20 and 30% extruded to 60 and 150 rpm.

With the aim of investigating the effect of plasticizer content on the PLA molecular structure, Cole–Cole diagrams were used (Figure 4). These diagrams are obtained by plotting the imaginary viscosity (η″) against the dynamic viscosity (η′) [38]. The Cole–Cole graph of a homogeneous material with a melt relaxation behavior described by a single relaxation time will form a semicircle [39]. On the other hand, in multiphase materials with several process and different relaxation times, the shape of the Cole–Cole graphs will be modified [39,40].

Cole–Cole plots show that PLA and PLA-ATBC blends are homogeneous systems with a concave shape (semicircles) and a single relaxation time, which indicates the compatibility between ATBC and the PLA matrix. Moreover, it is observed that plasticizer content decreases the elastic component of the viscosity and shortens the PLA relaxation times for the extrusion speeds studied. This behavior is related to an increase in the molecular mobility of PLA chains due to ATBC addition and increases dramatically with ATBC contents higher than 20% and high processing speeds.

**Figure 5:**
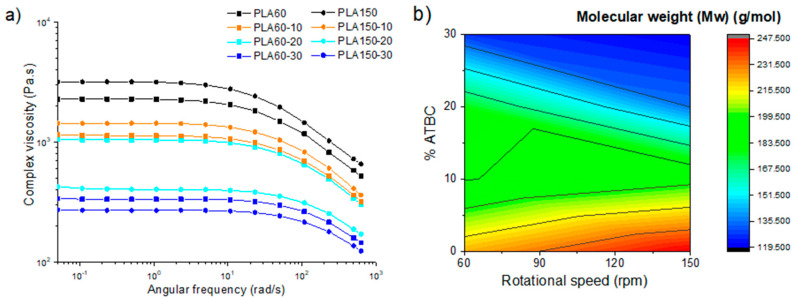
Rheological characteristics: (**a**) complex viscosity and (**b**) molecular weight calculated from the rheological data of PLA and PLA with ATBC at 10, 20 and 30% extruded to 60 and 150 rpm.

**Figure 6:**
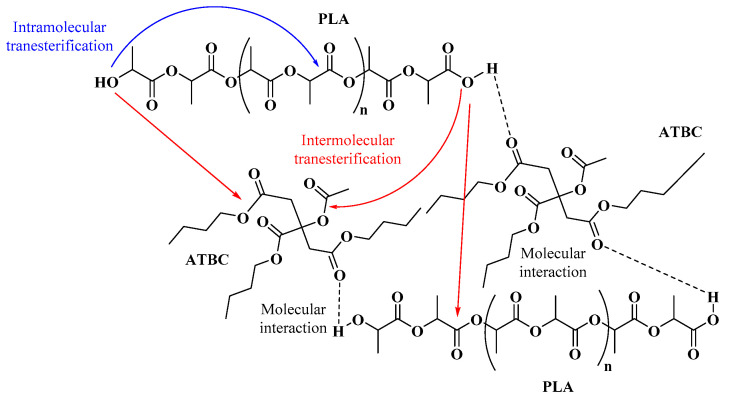
Proposed transesterification reactions and molecular interactions.

The complex viscosity (η*) values obtained (Figure 5a) for the mixtures were dependent on the proportion of plasticizer. As the amount of plasticizer increases, the η* decreases. For PLA60 and PLA60-10, lower viscosity values are observed with respect to the 150 rpm counterparts; however, the PLA150-20 and PLA150-30 samples exceed the values found in the 60 rpm counterparts. It can be inferred that plasticizer contents greater than 20% promote the decrease in molecular weight, and this effect becomes more noticeable when processed at high speeds. Molecular weight values (Figure 5b) were estimated from rheological data based on theoretical principles [36] not calculated experimentally. Here, significant molecular weight loss of up to 25% is related to PLA plasticized with 20% ATBC from 178,000 to 135,000 g/mol at 60 and 150 rpm, respectively (Supplementary Materials Table S1). Studies in PLA without plasticizer showed no incidence of critical process variables, allowing processing in wide ranges of speed (50–250 rpm) and temperature (180–220 °C) without affecting the molecular weight and thermomechanical characteristics of PLA [7]. However, plasticization of PLA with ATBC under higher shear conditions shows appreciable changes in viscosity and consequently in molecular weight. This degradation in PLA during processing in the presence of plasticizers with ester groups may be due to transesterification reactions (Figure 6) [9,30,40]. 

In general, these polymers show a typical shear-thinning behavior, this dependence against the frequency range studied was very noticeable mainly in the PLA60. The decrease in complex viscosity denotes greater molecular mobility. This relationship was also corroborated with the behavior of the modulus (G′ < G″), and these variables are indirectly related to increased heat capacity (C_p_), which leads to low thermal diffusivity in the material [41].

### 3.2. FTIR Analysis 

**Figure 7:**
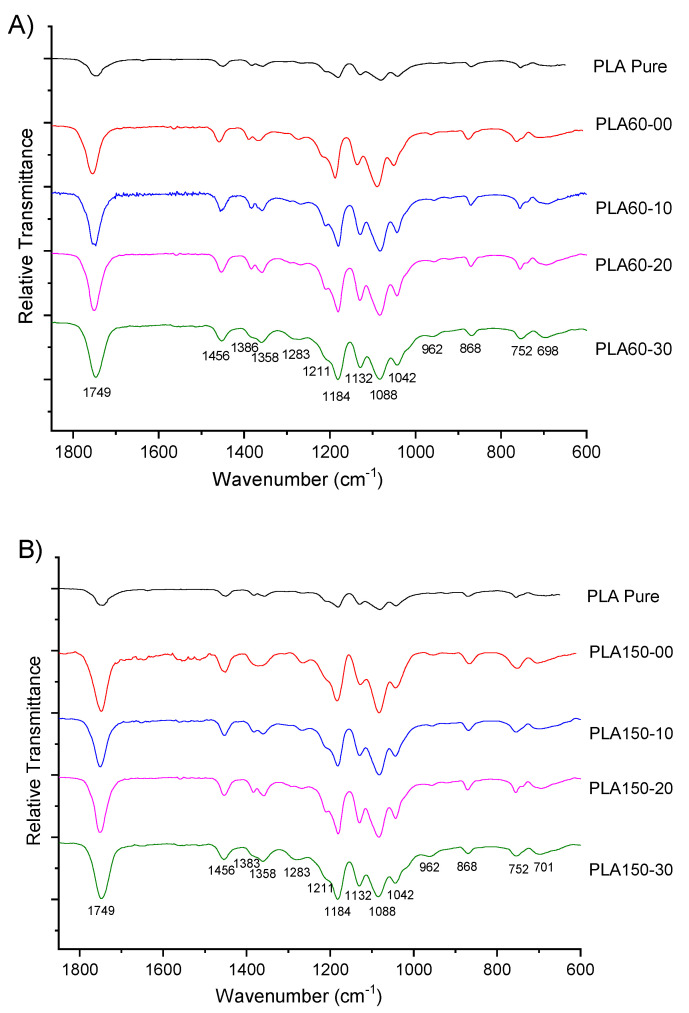
FTIR spectra of the PLA and PLA/ATBC samples: (**A**) 60 and (**B**) 150 rpm.

The FTIR spectra of PLA pellets, neat PLA and plasticized PLA are shown in Figure 7 and Figure S1. A couple of bands around of 2995 and 2950 cm^−1^, corresponding to C-H aliphatic symmetric and asymmetric stretching vibrations [26,42], increase with the amount of ATBC. At 1750 cm^−1^, the carbonyl stretching band appears [13,43], showing a rise in the intensity because of the ATBC and PLA interactions [27]. An absorption band centered at 1455 cm^−1^ of the -CH_3_ group decreases when the ATBC relation increase [26]; while the bands centered at 1455 cm^−1^, 1358 cm^−1^ and 1212 cm^−1^ become wider [27,42]. The typical absorption of the C-O asymmetric stretching in the ester group appear centered at 1182 cm^−1^ [19] and also decrease for the ATBC interaction [27]; finally, the bands at 868 and 754 cm^−1^ correspond to the amorphous and crystalline phases of PLA, indicating a semi crystalline plasticized PLA [13,26].

Accordingly, when the ATBC relation increased in the blend, some characteristic bands became broader due to an efficient interaction between the carboxylic groups through hydrogen bonds (Figure 5) [26,27]; furthermore, the terminal carboxylic groups are increasing in the blend because the melt mixing conditions, such as temperature, shearing rate, and length of residence, reduce the PLA molecular weight as a consequence of the susceptibility to the moisture and transesterification reactions [5,40]; for example, the PLA processing with ATBC decrease the molecular weight of the biopolymer by half at 60 rpm and 20% ATBC [43].

### 3.3. Thermal Analysis

The results of the thermogravimetric analysis are presented in Table 1, and the thermograms (Figures S2 and S3) showed that plasticized samples with ATBC and processed at lower extrusion rates have higher thermal stability compared to plasticized samples at higher speeds (150 rpm). In general, the samples of PLA and plasticized PLA show the temperature of maximum decomposition (T_max_) between 362.6 and 369.7 °C, where we found no significant differences. In the same way, the cutting temperatures were around 380 °C. However, the temperatures dropped by 10% by weight (T_10_). As the percentage of plasticizer increases, the T_10_ falls by ~30%, this behavior being more noticeable in the samples processed at 150 rpm. The above, due to the heat generated by the severe shear conditions, facilitates the evaporation of the plasticizer, which is evidenced at 224 °C (see T_10_, Figure S2c). The behavior of the mixtures is largely related to the extrusion speed used in this study, so this knowledge could contribute to obtaining future materials in which different conditions will be used in the process, knowing how speed influences function of the proportion of plasticizers more convenient to mix the PLA. Some studies report the extruded behavior of PLA at different speeds and process temperatures; however, the addition of plasticizer has not been considered [7]. 

Regarding melting temperatures and enthalpies (Figure S4), it was observed that the PLA/ATBC samples favored the transformation process by presenting a lower heat requirement compared to PLA. Incorporation of ATBC at a speed of 60 rpm achieves a radical change in the heat absorption of the solid–liquid transition with low enthalpy values (≤30 J/g).

The glass transition temperatures of the mixtures made at 60 rpm are lower than the T_g_ of the PLA without additive processed at 60 rpm and are in turn lower than that of the raw PLA (T_g_ = 62.2 °C). In terms of efficiency as plasticizers for PLA, it was observed that a higher content of plasticizer promotes lower T_g_ values. In contrast, extrusion speeds had a lower incidence, slightly presenting a decrease in T_g_ with low extrusion speeds. Dynamic mechanical analysis, on the other hand, is known to have higher sensitivity when it comes to detecting phase separation in mixtures, and, therefore, DMA measurements were made to further investigate the properties of the material in the shaped film. This type of study has been carried out in mixtures of PLA with different plasticizers prepared in a Brabender equipment, where a linear decrease in T_g_ was observed with the gradual increase of 10% in the concentration of the plasticizer; this was reduced from 54 to 30 °C, from neat PLA to PLA with 30% ATBC, respectively [44]. However, these mixtures presented the migration of the plasticizer due to the signs of phase separation, so it was not feasible to obtain results of mechanical resistance or barrier to analyze the applicability of the material.

The enthalpies of crystallization of the material processed at 60 rpm show a downward behavior, going from 27.4 J/g for PLA without additive to 18.8 J/g for PLA added with 30% ATBC; while for the material processed at 150 rpm the enthalpies have a minimum value for PLA without additive of 25.7 J/g and a maximum value of 17.8 J/g for PLA added with 30% ATBC. The behavior related to the melting enthalpy values showed similar trends because with a said increase in plasticizer the enthalpy values decrease; however, the increase in velocity caused these values to be higher, going from 30.7 J/g at 33.2 J/g for 60 and 150 rpm, respectively. These results are related to those found by Hassouna et al. [14]. The crystallinity values of the different mixtures that incorporate 20% and 30% of plasticizer achieve values equal to or greater than those obtained in the extruded material without plasticizer. In all cases, the percentage of crystallinity decreased dramatically, the effect being more marked in the samples processed at a slower speed, these results are comparable to those found by Mysiukiewicz et al. [7]. The second peak in melting temperature can be attributed to the lower stability of crystals that undergo macromolecular rearrangement during heating [45].

In general, the glass transition, crystallization, and melting temperatures are observed to be decreased, being more affected by shear than by residence time, two parameters dependent on the speed of processing. In the rheological analysis, structural change can be corroborated with the thinning of the material. As we can see, the PLA/ATBC samples register evaporation of the plasticizer at temperatures greater than 200 °C, a processing condition that was taken into account for the development of these materials.

### 3.4. Tensile Properties

**Figure 8:**
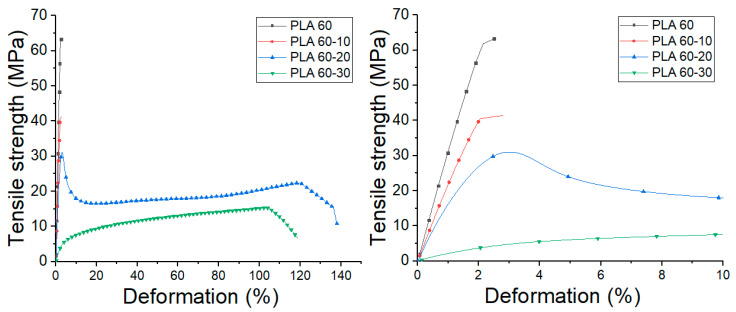
Average tensile stress vs. deformation PLA and PLA-ATBC processed at 60 rpm.

**Figure 9:**
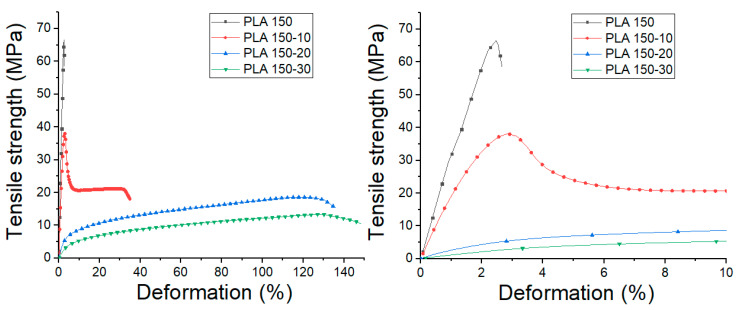
Average tensile stress vs. deformation PLA and PLA-ATBC processed at 150 rpm.

**Figure 10:**
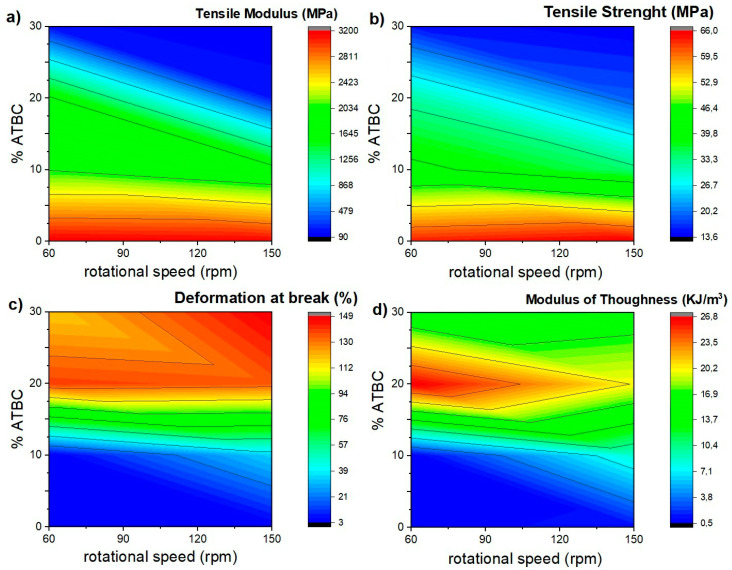
Two-dimensional contour of neat PLA and PLA-ATBC tensile properties: (**a**) Tensile Modulus, (**b**) Tensile Strength, (**c**) Deformation at Break, (**d**) Modulus of Toughness.

The influence of extrusion speed and ATBC content on the tensile properties were evaluated. The stress vs. strain graphs obtained from the tensile tests of each material are shown in Figure 8 and Figure 9, and the mechanical properties with the statistical study performed are summarized in Table 2. Moreover, the obtained results were presented in two-dimensional contour graphs (Figure 10). 

From these results, it is observed that processing speed does not generate significant changes in the tensile modulus (TM), maximum strength (TS), the deformation at break (εb) and toughness modulus of neat PLA (*p* value ≥ 0.05). On the other hand, the addition of ATBC in the percentages studied generates significant changes in the mechanical properties of the PLA. In general terms, a low molecular weight plasticizer like ATBC behaves like a solvent and generates a reduction in the cohesion between the polymer chains penetrating between them and decreasing the cumulative intermolecular forces between the polymer chains. 

With the addition of 10% ATBC, the TM values decrease by 36 and 44% compared to neat PLA for 60 and 150 rpm. Likewise, TS values decrease by 36 and 50%, respectively, due to the lower molecular adhesion between polymeric chains. Moreover, a substantial increase in εb and toughness is observed in samples processed at 150 rpm. For PLA 60-20 and 150-20 blends, TM values decrease by 47 and 93%, while TS decreases by 50 and 72% compared to neat PLA. Likewise, an increase in εb is observed up to 138%, and the highest toughness values are reached in the studied mixtures. With the addition of 30% ATBC, the decrease in the values of TM and TS continue to decrease up to 97 and 80%, respectively, compared to neat PLA, while εb increases up to 148%. However, the toughness values, obtained from the measurement of the area under the stress vs. strain graphs, decreases in comparison with PLA-ATBC 20% blends.

At this point, it is important to highlight that TM and TS values of all PLA-ATBC blends processed at 60 rpm are significantly higher (*p* < 0.05) than those obtained using 150 rpm, which could be related to the greater drop in molecular weight observed in blends processed at higher extrusion speeds. These results are important bearing in mind that the aim of a plasticizer incorporation is to improve the deformation capability of the polymeric matrix and reduce it brittleness while the mechanical strength and toughness are maintained. This combination of properties was achieved in the mixtures with a percentage of ATBC of 20% processed at 60 rpm. This result could generate benefits in the processing parameters optimization of plasticized PLA in the search for the application of this biodegradable material in product development using large volume manufacturing processes such as extrusion and thermocompression [46]. On the other hand, it is important to note that tensile properties obtained in our study by thermocompression are slightly lower than those obtained in PLA-ATBC blends obtained by injection molding [16]. A possible explanation for this could be the difference in orientation induced in the material during injection and thermocompression processes. During the injection molding process, the molten polymer is subjected to extensional flow fields. This technique has been shown to preferentially align polymer chains in the flow direction, increasing stiffness and strength as a function of extensional flow intensity. On the other hand, in processes such as thermocompression, the material is molded by the action of temperature and pressure, without subjecting the material to large shear stresses or extensional flow, for which the preferential alignment is almost non-existent [44,47].

### 3.5. Dynamic Mechanical Analysis

**Figure 11:**
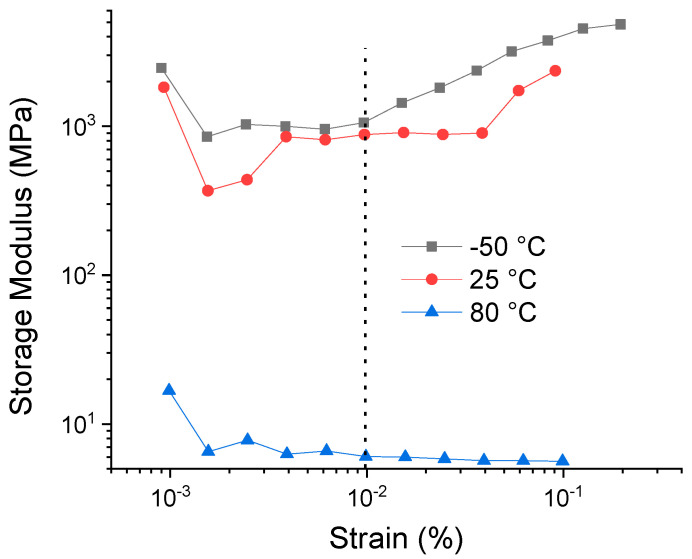
Strain sweep for PLA-60 at −50, 25 and 80 °C.

Figure 11 shows the results of the deformation sweep of the PLA 60 sample at −50, 25 and 80 °C, respectively. These runs were carried out with the aim of locating the linear viscoelastic region (LVE) of the studied materials. These results show a decrease in the values of the storage modulus (E′) with the temperature, which is related to a softening of the PLA matrix. Likewise, it is observed that the LVE is stable at a deformation of 0.01% at the studied temperatures, so this deformation was selected for the temperature ramp tests, from which the changes in the storage module were evaluated (E′), loss module (E″) and Tan δ (loss factor).

**Figure 12:**
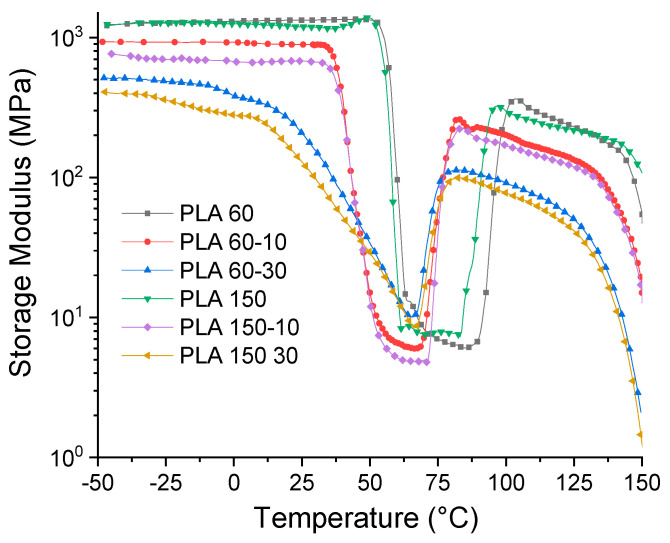
Storage modulus for PLA and PLA/ATBC.

Figure 12 shows the behavior of E as a function of temperature. It is observed that the values of E′ and the shape of the curve of the neat matrices processed at 60 and 150 rpm are very similar. These present an initial zone between −25 and 50 °C, where E′ gradually decreases with increasing temperature, which is related to the beginning of the softening of the polymer matrix. In the second zone located between 50 and 60 °C, there is a drastic decrease in the values of E′, which indicates that the molecular movements of the PLA chains increase when the T_g_ of the material is exceeded. Subsequently, between 75 and 100 °C, there is an increase in the values of E′, which is generated by a molecular rearrangement that generates the formation of crystals during heating or the cold crystallization process observed in the DSC tests. Finally, a last zone of decrease in E′ values is generated with temperature, increasing the mobility of the polymer chains. On the other hand, it is observed that the addition of ATBC generates a change in the shape of the E′ curve, and the drop in the E′ values begins at temperatures between 12 and 15 °C; this is associated with the decrease in the cohesive forces between polymer chains due to the effect of the plasticizer. Likewise, it is observed that the rigidity of the PLA matrix is proportional to the ATBC content and to the processing speed. The higher the ATBC content and the higher the extrusion speed, the lower the E′ values. These results are related to the effect of the plasticizer on the mobility of the PLA chains and are consistent with the results obtained by the tensile tests (Section 3.4). Table 3 summarizes the E′ values of the different materials at different temperatures.

Brostow et al. demonstrated that, within the materials universe, there exists a relationship between the resistance to deformation of a material, which is related to E′, the deformation at break (εb) and the brittleness (B) [48]. This approach uses the results obtained in the quasi-static tensile tests and dynamic mechanical tests and verifies that a material with a high deformation at break will be less brittle. These researchers also observed an inverse relationship between B values and the modulus of toughness of several materials (including metals and polymers), which is defined as the area under the stress vs. strain curve determined in the tension test [49]. Our results show that the PLA brittleness (B) tends to decrease with plasticizer addition plasticizer due to the increase in the deformation at break (see Table 2 and Table 3). It is also observed that the modulus of toughness (See Table 2) presents an inverse relationship with brittleness (B). These results show that ATBC addition decreases the PLA brittleness and increases the absorbed energy until break (failure).

**Figure 13:**
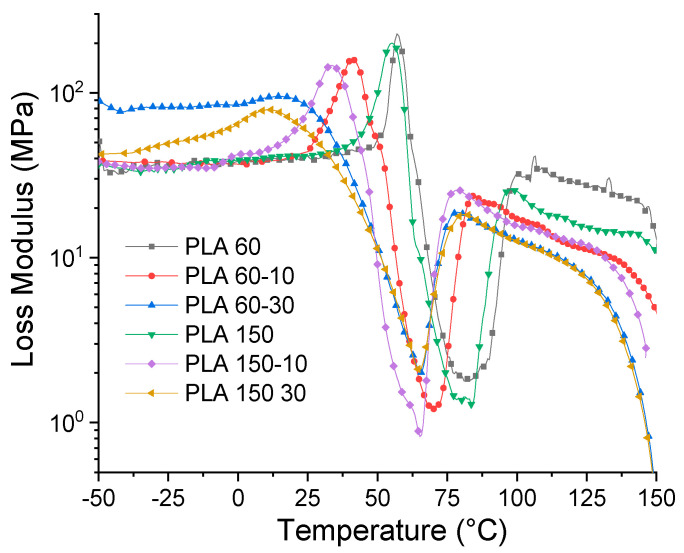
Loss modulus for PLA and PLA/ATBC.

Figure 13 shows the dependence of E″ on temperature for neat and plasticized PLA at different processing speeds. The E″ curves of neat PLA exhibit single peaks between 55 and 57 °C at 69 and 72 °C for PLA-60 and PLA-150, respectively, which are related to the start of movement of the PLA chains and reflects the process glass transition in the amorphous phase. The E″ graphs of the PLA-ATBC 10% mixtures show wider peaks, lower peaks, and shifted towards lower temperatures compared to the PLA samples. This trend is greater with the increase in ATBC content; the higher the ATBC content and processing speed, the shorter and wider the E″ peak. This phenomenon indicates that the addition of plasticizer and the increase in the processing speed increases the mobility of the polymer chains due to the plasticizing effect of ATBC and possibly due to the chain cut generated with the increase in the processing speed observed in the decrease of molecular weight (Section 3.1).

**Figure 14:**
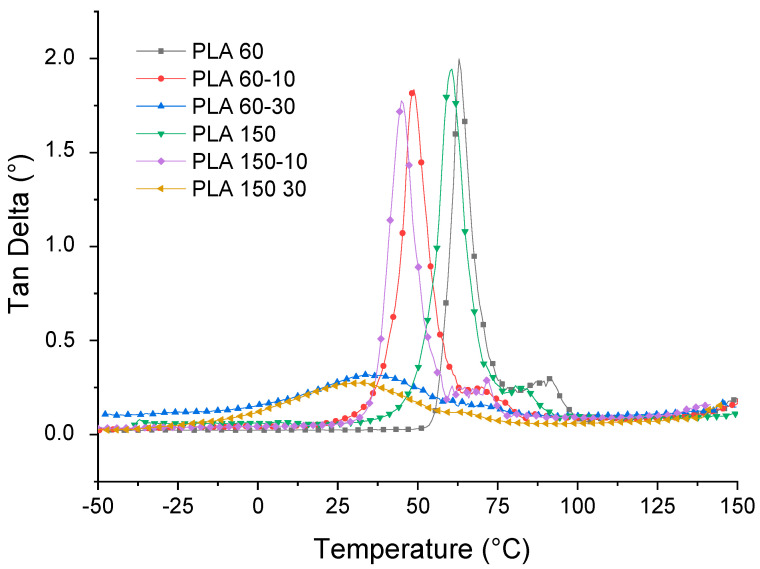
Tan delta for PLA and PLA/ATBC.

The variation of Tan δ with temperature is shown in Figure 14. This property is defined as the relationship between the loss and storage modules and is related to the damping properties of a material. The results show that the glass transition temperature (T_g_) decreased significantly for all the plasticized PLA samples proportional to the plasticizer content and the processing speed.

For example, at 10% ATBC, the glass transition of PLA decreases from 63 to 48 °C for samples processed at 60 rpm and from 60 to 44 °C for samples processed at 150 rpm, respectively. With the increase in the percentage of ATBC, glass transition continues to decrease to 35 and 31 °C for samples processed at 60 and 150 rpm, respectively. These glass transition temperature (T_g_) values obtained by DMA are similar to those reported in the literature for PLA-ATBC [26] and PLA-PBAT-ATBC mixtures [50] and have the same trend as the results found by the DSC (see Table 1), where T_g_ decreases due to the addition of plasticizer.

In order to further understand the effect of processing speed on ATBC dispersion within the PLA matrix, the measurement of the Full width at half maximum (FWHM) of Tan δ peaks was performed. Such a larger δ peak generates a higher FWHM value and implies more interaction and contact between the phases of the mixture, which generates materials with a wide range of relaxation times [39,51]. In addition, large FWHM is related to low dispersion and a heterogeneous blend. The results show that the FWHM increases with the addition of the plasticizer and with the processing speed, which means that the mixtures obtained at 60 rpm have greater homogeneity and confirms that the plasticizer is better dispersed within the PLA matrix. 

### 3.6. Scanning Electron Microscopy Analysis

**Figure 15:**
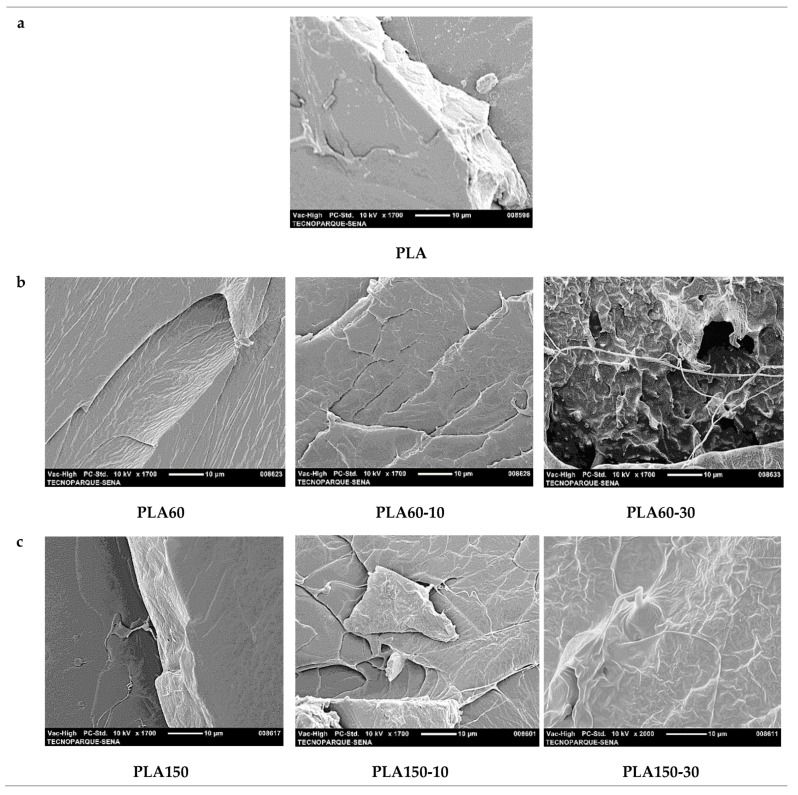
Micrographs obtained by scanning electron microscopy (SEM) with magnifications of 1700×for (**a**) PLA, (**b**) PLA60, PLA60-10 and PLA60-30, (**c**) PLA150, PLA150-10 and PLA150-30.

Figure 15 and S6 shows the SEM micrographs of the most representative samples. The typical brittle fracture morphology of neat PLA can be seen in Figure 15a. Samples incorporating ATBC at 60 rpm (Figure 15b) exhibited a rougher surface. Incorporating ATBC shows filaments and some microcavities that extend directly under the impact load of the samples. In the PLA sample processed at the highest extrusion speed (150 rpm, Figure 15c), a continuous and rough phase morphology was observed, just like the neat PLA (see Figure S5, micrographs with magnifications of 500x). The above phenomena indicated that the PLA/ATBC mixtures belonged to a partially compatible system, and the ATBC was constantly dispersed in the PLA matrix in a state of spherical droplets. Comparison of the tensile cross-section micrographs of the PLA mixtures plasticized with 10% to 30% by weight ATBC and the neat PLA shows that the dispersed ATBC phase contributes to the ductility of the material, the induction of filaments that show a decrease in crystallinity under tensile stresses at 60 rpm, compared to 150 rpm, being more noticeable.

### 3.7. Contact Angle Analysis

**Figure 16:**
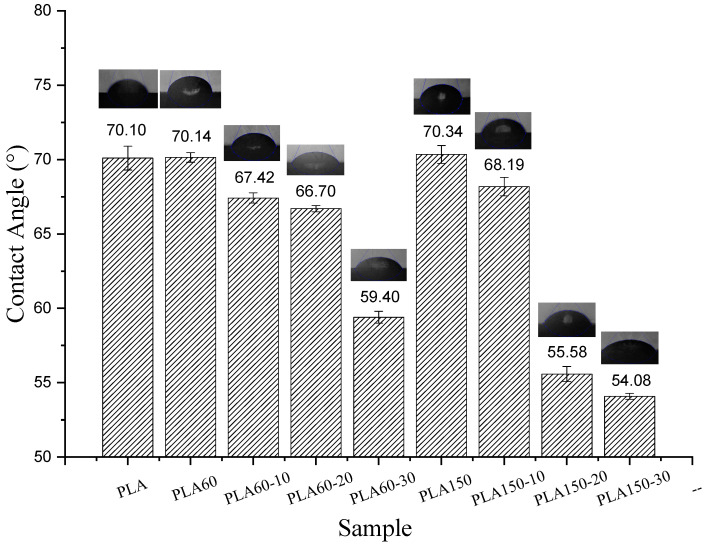
Average contact angle values for PLA and PLA/ATBC with different proportions.

The contact angle values of neat PLA were higher (70.1°) compared to the PLA/ATBC mixtures (Figure 16); this was to be expected since the plasticization introduced polar molecules into the PLA corresponding to functional groups (C-O and C=O) that promote the generation of intermolecular interactions through hydrogen bonds with water. The results obtained are consistent with the percentage of ATBC incorporation by these samples, which, with this increase, reduced the contact angle proportionally. Therefore, the hydrophilicity was increased. The results obtained are important since it was possible to obtain a reduction in the contact angle of up to 15.2% and 22.8% of the PLA60-30 and PLA150-30, respectively. This can greatly enhance the effectiveness of this material as a compatibilizing agent for PLA/hydrophilic polymer blends. It is observed that the mixtures developed at higher speeds do not allow the polar ATBC molecules to be incorporated within the polymer chain. Being exposed on the surface of the mixtures, these phenomena have been discussed in other biopolymer systems with plasticizer [52]. Additionally, plasticized blends have increased the polymer chain mobility as a consequence of the loss of molecular weight [27]; therefore, the blend obtained at 150 rpm shows a greater diffusion process. In previous research, several authors have mentioned that PLA plasticized with ATBC improves compatibility in multiphase systems, especially when the proportion of starch is less than 60% in which this type of mixture reaches a maximum saturation. [53]. However, mixing conditions should be considered in which the plasticizer penetrates the polymer chains and reduces the hydrophilicity of the final material.

## 4. Conclusions

Morphology, thermal, mechanical, and dynamic mechanical properties, as well as surface characteristics were investigated. Scanning electron microscopy (SEM) micrographs of the compounds show intimate contact at both speeds, with the presence of filaments leading the tension at the moment of fracture being more noticeable. This enhanced interaction was confirmed by the results of Fourier transform infrared spectroscopy (FTIR) that show the presence of a hydrogen-bonding interaction between PLA and ATBC. Thermal stability (as determined by thermogravimetric analysis, TGA) at both speeds decreased with increasing plasticizer content; nonetheless, the higher process speed (150 rpm) caused a radical decrease in temperature at 10% loss by weight (T_10_) up to 100 °C with 30% ATBC. Differential scanning calorimetry (DSC) results did not show an influence of the plasticizer on the melting characteristics of PLA; however, the influence of the plasticizer was significant on cold crystallization and the melting behavior of PLA, even with the low ATBC content of 10–30%. Both the storage modulus and the PLA loss modulus decreased with increasing PLA plasticizer content. At a higher rate (150 rpm), viscous to elastic transitions are observed at lower frequency ranges, compared to samples processed at 60 rpm. The tensile test results show that ATBC addition improves the deformation capability (up to 148%) and toughness of the polymeric matrix, reduces it brittleness and ensures that the mechanical strength is maintained (up to 97 and 80%, respectively). The best mechanical performance was achieved in the PLA-ATBC blends with a percentage of ATBC of 20% processed at 60 rpm. The results of the dynamic mechanical analysis (DMA) show that ATBC content and the extrusion speed influence the E′ values of the PLA matrix. The obtained results are related to the effect of the plasticizer on the mobility of the PLA chains and are consistent with the results obtained by the tensile tests. Moreover, the analysis of the broadness of Tan peaks shows that PLA-ATBC blends obtained at 60 rpm have greater homogeneity and confirms that the plasticizer is better dispersed within the PLA matrix.

## Figures and Tables

**Table 1:** Thermal properties of PLA and PLA/ATBC.

Sample	TGA		DSC					
	T_10_	T_max_	T_g_	T_cc_	T_m_	ΔH_m_	ΔH_cc_	%X_c_
	°C					J/g		
**PLA**	342.8	367.4	62.2	103.3	150.6	44.2	33.4	11.6
**PLA60-00**	339.3	367.6	58.6	103.6	151.3	30.7	27.4	3.5
**PLA60-10**	302.1	365.1	41.0	93.4	145.4	25.6	22.4	3.1
**PLA60-20**	280.9	363.8	37.3	90.4	143.5	25.4	21.6	3.3
**PLA60-30**	262.4	362.6	32.1	76.7	137.6	24.2	18.8	4.1
**PLA150-00**	343.1	369.7	52.4	111.4	157.8	33.2	25.7	8.1
**PLA150-10**	315.2	367.2	43,4	92.2	145.4	27.0	23.6	3.3
**PLA150-20**	274.1	366.3	39.3	80.0	139.0	27.1	22.4	4.0
**PLA150-30**	239.6	364.5	38.2	72.0	135.3	25.4	17.8	5.7

**Table 2:** Mechanical properties of the studied materials.

Mechanical Properties	Sample	60 rpm *	150 rpm *
Tensile Modulus (MPa)	PLA	3198 ± 132 ^a^	3162 ± 96 ^a^
	PLA-10	2020 ± 137 ^a^	1774 ± 111 ^b^
	PLA-20	1678 ± 97 ^a^	203 ± 23 ^b^
	PLA-30	176 ± 22 ^a^	98 ± 10 ^b^
Tensile Strength (MPa)	PLA	64.0 ± 2.7 ^a^	66.9 ± 2.0 ^a^
	PLA-10	41.2 ± 4,5 ^a^	34.2 ± 2.2 ^b^
	PLA-20	31.8 ± 3.1 ^a^	18.6 ± 1.3 ^b^
	PLA-30	15.5 ± 1.0 ^a^	13.7 ± 1.3 ^b^
Deformation at break (%)	PLA	2.5 ± 0.1 ^a^	2.7 ± 0.1 ^a^
	PLA-10	2.8 ± 0.1 ^a^	34.2 ± 14.8 ^b^
	PLA-20	138.0 ± 16.0 ^a^	134.6 ± 7.3 ^a^
	PLA-30	118.6 ± 9.5 ^a^	148.4 ± 11.3 ^b^
Modulus of Toughness (kJ/m^3^)	PLA	0.9 ± 0.5 ^a^	1.2 ± 0.1 ^a^
	PLA-10	0.6 ± 0.1 ^a^	8.4 ±2.6 ^b^
	PLA-20	26.8 ± 3.9 ^a^	20.1 ± 2.6 ^b^
	PLA-30	14.3 ± 1.6 ^a^	15.5 ± 2.5 ^a^

^a–b^ Different letters in the same file of each mechanical property indicate significative differences (*p* < 0.05). * Mean of five replications ± standard deviation.

**Table 3:** Dynamic Mechanical Analysis (DMA) results of the studied materials.

Sample	E′ (MPa)			Brittleness (Pa.%/10^10^) *	T_g_ (°C) **	Full Width at Half Maximum (FWHM) of Tan δ Peaks ***
	−25 °C	25 °C	100 °C			
**PLA60-00**	1266	1327	320	3	63	7.1
**PLA60-10**	928	888	198	4	48	9.2
**PLA60-30**	487	211	89	0.4	35	38.4
**PLA150-00**	1271	1196	288	3.1	60	8.3
**PLA150-10**	698	685	167	0.4	44	11.5
**PLA150-30**	360	130	76	0.5	31	45.5

* Brittleness B = 1/ ε_b_*E′. ** T_g_ values were taken at the maximum peak of Tan delta curves. *** FWHM values were taken after a baseline correction of Tan delta curves.

## Supplementary Materials

The following are available online at https://www.mdpi.com/2073-4360/12/9/2111/s1, Figure S1: IR spectra of the PLA and PLA/ATBC samples (A) 60 rpm and (B) 150 rpm. Figure S2. TGA thermograms of the samples of (a) PLA with ATBC at 10, 20 and 30% extruded to 60 rpm. (b) PLA with ATBC at 10, 20 and 30% extruded to 150 rpm. Figure S3: Derivative TGA curves of the samples of (a) PLA with ATBC at 10, 20 and 30% extruded to 60 rpm. (b) PLA with ATBC at 10, 20 and 30% extruded to 150 rpm. Figure S4: DSC thermograms of the samples of (a) PLA with ATBC at 10, 20 and 30% extruded to 60 rpm. (b) PLA with ATBC at 10, 20 and 30% extruded to 150 rpm. Figure S5: Micrographs obtained by SEM with magnifications of 500x for (a) PLA, (b) PLA60, PLA60-10 and PLA60-30, (c) PLA150, PLA150-10 and PLA150-30. Table S1. Zero shear viscosity, characteristic relaxation time and molecular weight from the rheological data of PLA and PLA with ATBC.
