# Supplementary material for: Effect of Extrusion Screw Speed and Plasticizer Proportions on the Rheological, Thermal, Mechanical, Morphological and Superficial Properties of PLA
Source: Polymers (Basel). 2020 Sep 16;12(9):2111. doi: 10.3390/polym12092111 (PMC7570249; doi:10.3390/polym12092111)
Supplement: Supplementary file 1 [file polymers-12-02111-s001.pdf]

polymers

MDPI

Article

Effect of Extrusion Screw Speed and Plasticizer Proportions on the Rheological, Thermal, Mechanical, Morphological and Superficial Properties of PLA

Jaime Gálvez [1,†], Juan P. Correa Aguirre [2,†], Miguel A. Hidalgo Salazar [2], Bairo Vera Mondragón [1], Elizabeth Wagner [1] and Carolina Caicedo [3,*]

1 Grupo de Investigación en Desarrollo de Materiales y Productos—GIDEMP, Centro Nacional de Asistencia Técnica a la Industria—ASTIN, SENA, Calle 52 No 2bis 15, Cali 760035, Colombia; jagalvez39@misena.edu.co (J.G.); bvera@sena.edu.co (B.V.M.); ewagner@misena.edu.co (E.W.)
2 Research Group for Manufacturing Technologies (GITEM), Universidad Autónoma de Occidente, Cali 760035, Colombia; jpcorrea@uao.edu.co (J.P.C.A.); mahidalgo@uao.edu.co (M.A.H.S.)
3 Grupo de Investigación en Química y Biotecnología (QUIBIO), Facultad de Ciencias Básicas, Universidad Santiago de Cali, calle 5 No. 62-00, Cali 760035, Colombia
* Correspondence: carolina.caicedo03@usc.edu.co
† These authors contributed equally to this work and should be considered as co-first authors.

Received: 26 August 2020; Accepted: 8 September 2020; Published: date

Supporting Information

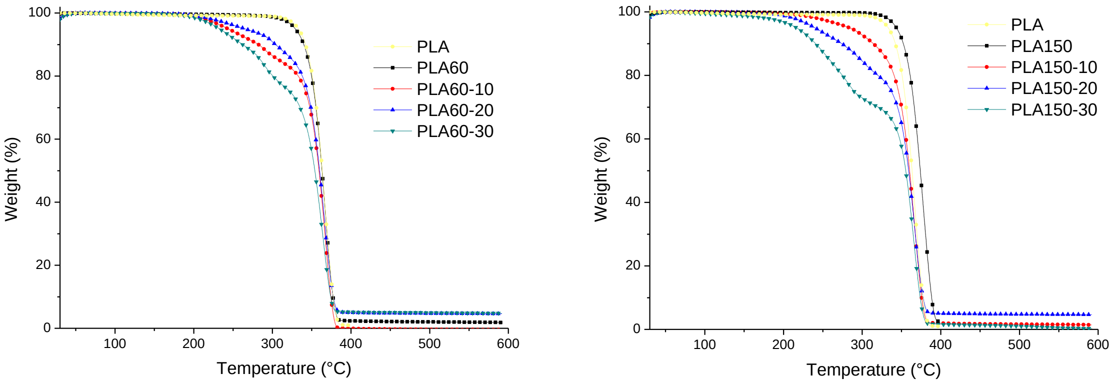

(a) (b)

Figure S1. TGA thermograms of the samples of (**a**) PLA with ATBC at 10, 20 and 30% extruded to 60 rpm. (**b**) PLA with ATBC at 10, 20 and 30% extruded to 150 rpm.

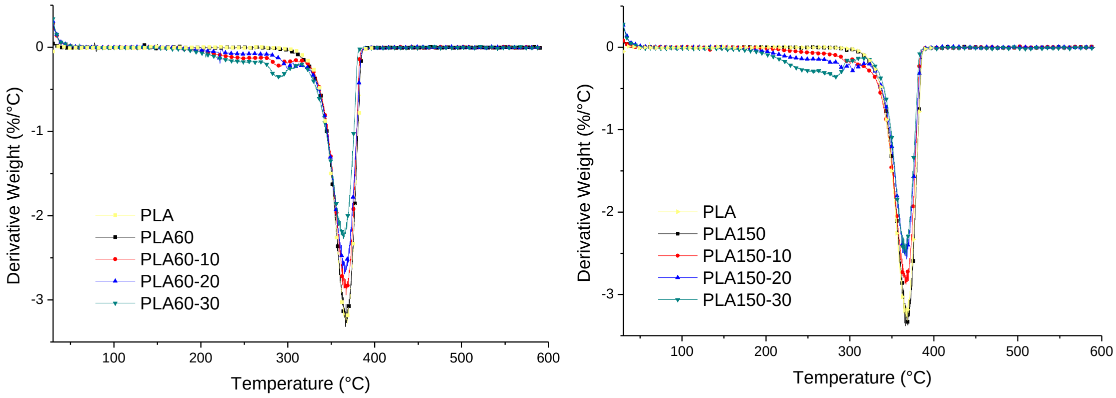

(**a**) (**b**)

Figure S2. Derivative TGA curves of the samples of (**a**) PLA with ATBC at 10, 20 and 30% extruded to 60 rpm. (**b**) PLA with ATBC at 10, 20 and 30% extruded to 150 rpm.

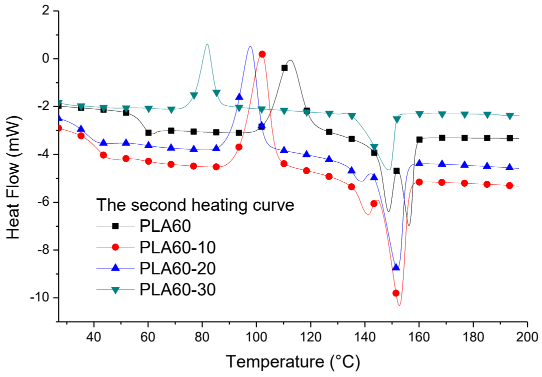

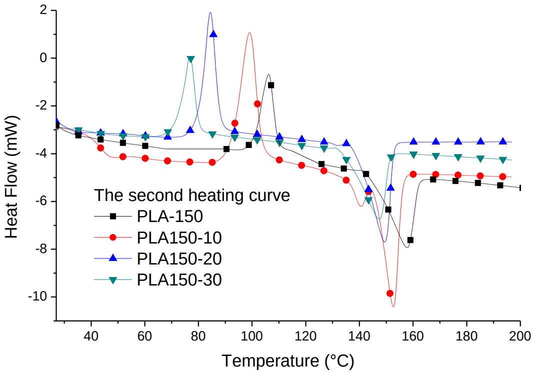

(a) **(b)**

Figure S3. DSC thermograms of the samples of (**a**) PLA with ATBC at 10, 20 and 30% extruded to 60 rpm. (**b**) PLA with ATBC at 10, 20 and 30% extruded to 150 rpm.

a

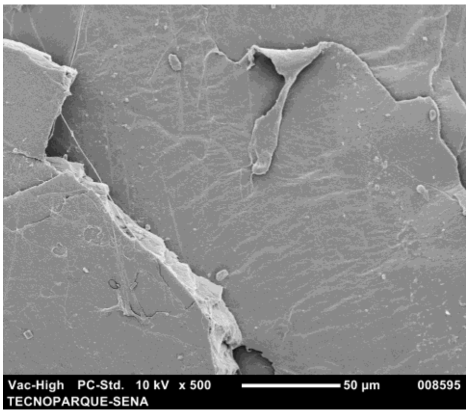

PLA

b

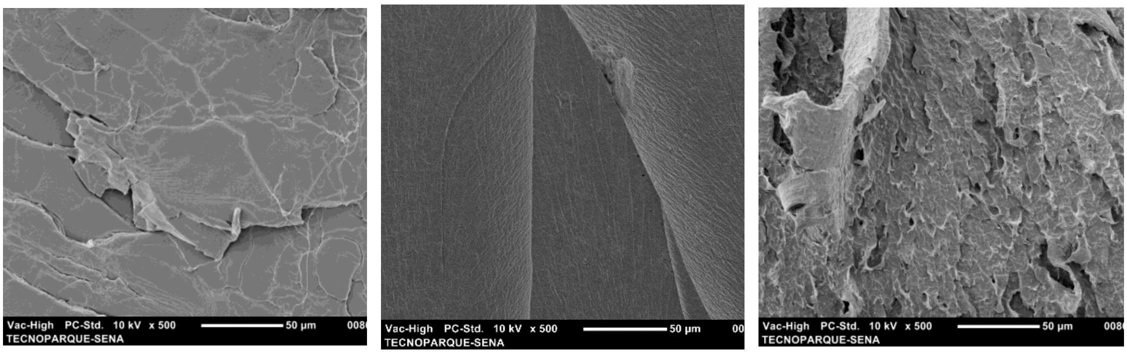

PLA60 **PLA60-10** **PLA60-30**

c

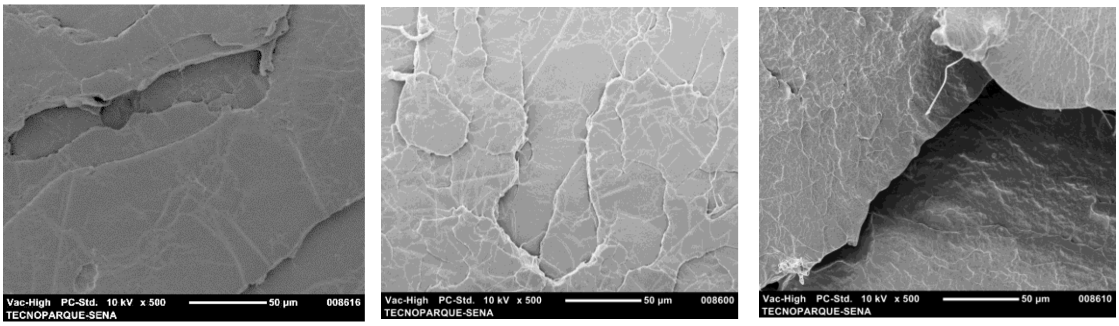

PLA150 **PLA150-10** **PLA150-30**

Figure S4. Micrographs obtained by SEM with magnifications of 500x for (**a**) PLA, (**b**) PLA60, PLA60-10 and PLA60-30, (**c**) PLA150, PLA150-10 and PLA150-30.

Table S1. Zero shear viscosity, characteristic relaxation time and molecular weight from the rheological data of PLA and PLA with ATBC.

Sample	η0	λ (s)	Mw
PLA60	2298,5	0,0288	224090,5
PLA60-10	1147,1	0,0126	182663,8
PLA60-20	1054,8	0,0134	178211,5
PLA60-30	337,5	0,0073	127457,8
PLA150	3202,7	0,0311	247058,6
PLA150-10	1446,2	0,0165	195542,5
PLA150-20	412,4	0,0027	135204,0
PLA150-30	272,4	0,0071	119677,3

Table S2. DMA results of the studied materials.

**Sample**	**E′ (MPa)**			**Tg (°C)***	**Full width at half maximum (FWHM) of tan δ peaks****
	**-25 °C**	**25 °C**	**100 °C**		
**PLA60**	1266	1327	320	63	7.1
**PLA60-10**	928	888	198	48	9.2
**PLA60-30**	487	211	89	35	38.4
**PLA150**	1271	1196	288	60	8.3
**PLA150-10**	698	685	167	44	11.5
**PLA150-30**	360	130	76	31	45.5

*Tg values were taken at the maximum peak of tan delta curves.

** FWHM values were taken after a baseline correction of tan delta curves.

© 2018 by the authors. Submitted for possible open access publication under the terms and conditions of the Creative Commons Attribution (CC BY) license (http://creativecommons.org/licenses/by/4.0/).
